# Water vapour is a pre-oviposition attractant for the malaria vector *Anopheles gambiae sensu stricto*

**DOI:** 10.1186/1475-2875-12-365

**Published:** 2013-10-11

**Authors:** Michael N Okal, Benjamin Francis, Manuela Herrera-Varela, Ulrike Fillinger, Steven W Lindsay

**Affiliations:** 1 icipe-Thomas Odhiambo Campus, Mbita, Kenya; 2 Disease Control Department, London School of Hygiene & Tropical Medicine, London, UK; 3 School of Biological and Biomedical Sciences, Durham University, Durham, UK

**Keywords:** *Anopheles gambiae*, Oviposition, Water vapour, Gravid mosquitoes

## Abstract

**Background:**

To date no semiochemicals affecting the pre-oviposition behaviour of the malaria vector *Anopheles gambiae sensu lato* have been described. Water vapour must be the major chemical signal emanating from a potential larval habitat, and although one might expect that gravid *An. gambiae s.l.* detect and respond to water vapour in their search for an aquatic habitat, this has never been experimentally confirmed for this species. This study aimed to investigate the role of relative humidity or water vapour as a general cue for inducing gravid *An. gambiae sensu stricto* to make orientated movements towards the source.

**Methods:**

Three experiments were carried out with insectary-reared *An. gambiae s.s.* One with unfed females and two with gravid females during their peak oviposition time in the early evening. First, unfed females and gravid females were tested separately in still air where a humidity difference was established between opposite ends of a WHO bioassay tube and mosquitoes released individually in the centre of the tube. Movement of mosquitoes to either low or high humidity was recorded. Additionally, gravid mosquitoes were released into a larger air-flow olfactometer and responses measured towards collection chambers that contained cups filled with water or empty cups.

**Results:**

Unfed females equally dispersed in the small bioassay tubes to areas of high and low humidity (mean 50% (95% confidence interval (CI) 38-62%). In contrast, gravid females were 2.4 times (95% CI 1.3-4.7) more likely to move towards high humidity than unfed females. The results were even more pronounced in the airflow olfactometer. Gravid females were 10.6 times (95% CI 5.4-20.8) more likely to enter the chamber with water than a dry chamber.

**Conclusions:**

Water vapour is a strong pre-oviposition attractant to gravid *An. gambiae s.s.* in still and moving air and is likely to be a general cue used by mosquitoes for locating aquatic habitats.

## Background

*Anopheles gambiae sensu stricto* and *Anopheles arabiensis* are the two major vectors of malaria in Africa. Their primary larval habitats are commonly described as small, temporary, open, sunlit pools [1,2], yet this is a gross oversimplification of the types of habitat actually colonized by these mosquitoes [3]. In reality, immature stages of both species can be found in an enormous diversity of aquatic habitats and it has been difficult to characterize these sites with precision [4-7]. Semi-permanent water bodies are frequently as productive or even more productive over time than the small rain-filled puddles that are often only abundant during the rainy season [5-7]. Nearly every type of water accumulation, apart from excessively polluted smelly water, may contain anopheline larvae [3-5,8-11]. The presence of larvae in a water body is thought to be the result of a combination of the egg-laying choice of gravid females that deposit their eggs in water and the survival of larvae in those habitats [12], although the cues that guide the gravid female’s choice are not well understood.

The attractiveness of field sites may be due to general characteristics and cues such as their relative position in relation to the resting site of gravid females, visual cues from these sites and the presence of water vapour plumes, as well as more habitat-specific chemical cues released from water bodies serving as semiochemicals which indicate the suitability of an aquatic habitat [1,13,14]. Although some putative semiochemicals have been suggested based on coupled gas chromatography-electroantennogram detection [15-17], to date, no semiochemical has been confirmed to affect the behaviour of gravid *An. gambiae s.l.* Water vapour must be presumed to be the major chemical signal emanating from a potential larval habitat and although one might expect that gravid *An. gambiae s.l.* detect and respond to water vapour in their search for an aquatic habitat, this has never been experimentally confirmed for this species.

The present study set out to investigate the role of water vapour in the pre-ovipositional behaviour of *An. gambiae s.s.* which results in arrival at potential oviposition sites [14]. Two separate choice tests were used: in the first test the response of unfed and gravid *An. gambiae s.s.* were compared using still air in cages connected to WHO bioassay tubes, in the second test gravid female responses were tested using moving air in a newly designed airflow olfactometer. In both systems *An. gambiae s.s.* were provided with a choice of moving towards an area of low or high humidity without visual cues or access to the water source.

## Methods

### Study site

The study was carried out at the International Centre of Insect Physiology and Ecology, Thomas Odhiambo Campus (icipe-TOC), Mbita, on the shores of Lake Victoria, Kenya (0° 26’ 06.19” S, 34° 12’ 53.13”E; 1,137 m above sea level). This area is characterized by an equatorial tropical climate with an average minimum temperature of 16°C and an average maximum temperature of 28°C. The area experiences two rainy seasons: the long rainy season between March and June and the short rainy season between October and December. The average annual rainfall for 2010-2012 was 1,436 mm (icipe-TOC meteorological station).

### Mosquitoes

Insectary-reared *An. gambiae s.s.* (Mbita strain) were used for all experiments. Five-days-old females were selected 30 minutes prior to the experiment from insectary colony cages where they had been kept in groups of approximately 300 males and 300 females in 30 × 30 × 30 cm netting cages and provided with 6% glucose solution *ad libitum.* These females never had a bloodmeal and are therefore referred to as unfed females. Gravid mosquitoes were prepared by transferring 150 female and 150 male mosquitoes, aged two days old, in 30 × 30 × 30 cm netting cages and provided with 6% glucose solution *ad libitum* at 25-28°C and a relative humidity between 68-75%. Saturated cotton towels, 50 × 25 cm in area, were folded and placed over the cages to avoid mosquito desiccation. Mosquitoes were starved from sugar for seven hours and allowed to feed on a rabbit for 15 minutes on day two and three post-emergence and rested for a further two days before use. Thus five-days-old gravid females were used for experiments.

### Water

For all experiments, piped non-chlorinated water pumped from Lake Victoria was used. The water was passed slowly through a locally made sand charcoal gravel filter for purification. Briefly, two 50 L buckets were placed on top of each other. The lower bucket’s lid contained a hole and the upper bucket’s floor was perforated with small holes for the filtered water to pass through to the lower bucket. The upper bucket contained three layers of gravel, activated charcoal and sand. Tap water was poured into the top of the upper bucket and run slowly through the layers. The aim was to remove large and small particles from the water including the majority of algae and bacteria. The purified water is referred to as ‘filtered tap water’.

In the two bioassays described below it is hypothesized that the tap water was attractive solely because of the presence of water vapour rather than because the water contained an attractive semiochemical. This assumption is based on a preliminary experiment, that was implemented comparing the oviposition response of *An. gambiae s.s.* to filtered tap water and double-distilled water. A description of the experiment and results can be found in Additional file 1. Gravid females did not have a significant preference for either filtered tap water or distilled water.

### WHO-tube bioassays

**Figure 1:**
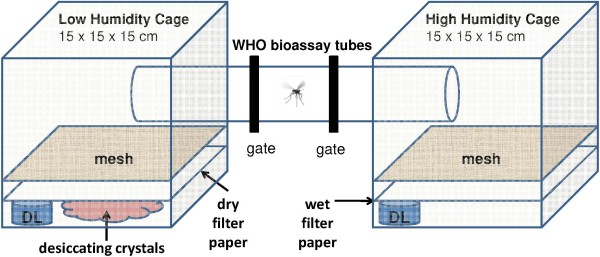
**WHO tube bioassays to observe response of individual gravid *Anopheles gambiae s.s. *towards high or low humidity.** DL = data logger.

Choice tests were carried out in the laboratory under ambient conditions. Natural moonlight came from a window located 2 m from the set-up. For each choice test, three WHO bioassay tubes, each 12 cm long [18] were connected together with open/close gates between the inner and outer tubes. The two outer tubes were inserted for approximately 6 cm into small mosquito cages measuring 15 × 15 × 15 cm. Cages were wrapped in commercially available kitchen cling-film (Figure 1). In one cage, 25 ml of silica gel desiccating crystals were spread evenly over the bottom of the cage, with dry filter paper covering the crystals. In the other cage, there were no desiccating crystals and the filter paper was dampened with 25 ml of filtered tap water. A 15 × 15 cm wire screen was fixed 5 cm above the bottom of the cages to prevent mosquitoes from making direct contact with the substrates. There were eight identical set-ups, arranged along a table 10 cm apart, with the high and low humidity ends being alternated between each set of tubes. In the first of these eight set-ups, data loggers (Tinytag, TV4500) were placed in the two cages to record the relative humidity. A single *An. gambiae s.s.* (Mbita strain) was placed in the middle tube at around 18.00 with the gates opened by 2 mm (not too wide to let the mosquito through) allowing some exchange of air within the central tube and the connected cages before the gates were completely opened at 18.30 allowing the mosquito to move freely from the tubes into the cages. This experiment was implemented with unfed and gravid females of the same age. At 19.00 the position of each mosquito either in the middle tube or in one of the two cages was recorded. The gates were closed at 21.30 and mosquitoes again counted in each cage or middle tube. The time period for observation was chosen based on preliminary experiments that have shown that out of 120 individual gravid females tested (5 round × 20 females) 95% (114/120) of the local insectary-reared *An. gambiae s.s.* (Mbita strain) laid all their eggs before 21.30, which is similar to the time reported for the same strain previously [19]. Experiments were done with eight mosquitoes each evening on nine occasions with unfed females and with gravid females (total 72 per physiological stage). During the experiment with unfed females four escaped when manipulating the gates and were excluded from the analyses, similarly when implementing the experiment with gravid females six females were found dead in the middle tube and were excluded from the analysis, therefore a total of 68 unfed and 66 gravid *An. gambiae s.s.* were tested. This sample size was sufficient to detect a 33% increase in the attractiveness of humid air (i e, 66.5% collected in the humid air cage compared with the 50% null hypothesis) at the 5% level of significance and 80% power (inference of a proportion compared to the null proportion [20]).

### Airflow olfactometer bioassays

**Figure 2:**
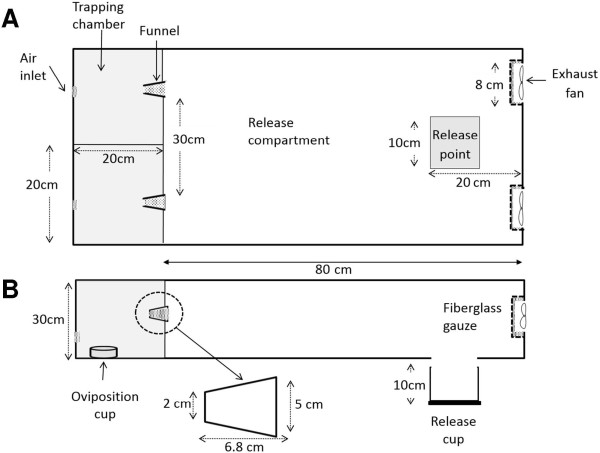
**Dual port airflow olfactometer.** View from the top **(A)** and view from the side **(B)**.

Three dual port airflow olfactometers were used to study the responses of gravid *An. gambiae s.s.* to filtered tap water (Figure 2). Each tunnel measured 40 × 100 × 30 cm and was made from polymethyl methacrylate sheets. Each tunnel was partitioned into three compartments: one large compartment for releasing the mosquitoes and two identical trapping chambers (20 × 20 × 30 cm each). Two fans (diameter 8 cm, 6 V computer casing fans (Molex, China)) drew air through the trapping chambers into the release compartment at 0.48 m/s. Batches of 100 gravid *An. gambiae s.s.* females were introduced at 18.20 by inserting a 10 × 10 × 10 cm cage into the underside of the release compartment. At the same time the fans were switched on. Mosquitoes acclimated for 10 minutes and were then released by carefully opening the cage at 18.30. Mosquitoes were able to fly through a transparent polyvinyl chloride funnel into a trapping chamber. Alternative trapping chambers of each tunnel were baited with either an empty 70 mm diameter glass cup (Pyrex®) or with the same type of cup filled with 100 ml of filtered tap water. Prior to any experiment glass cups were autoclaved and heated afterwards in an oven at 200°C for at least two hours to rid them of possible odorant contamination and bacteria. Mosquitoes trapped in the chambers and those that remained in the release compartment were counted at 08.00 the following morning. Experiments were done in complete darkness, at ambient conditions (27-28°C, 60-70% relative humidity) in a room without a window.

Responses of gravid *An. gambiae s.s.* were compared for three different treatments in an olfactometer: (1) both chambers contained dry cups, (2) both chambers contained cups filled with water, and (3) one chamber contained a dry cup (control) and the other a cup with water (test). In all cases cups were randomly allocated as ‘control’ or ‘test’ (even if the same treatments were provided) to the two chambers to help facilitate the analysis.

Each treatment was replicated 24 times (the ‘test’ cup of each treatment was located in each of the chambers of each of the three olfactometers four times) in order to estimate the variability in responses so that sample size calculations could be done. Power calculations were based on the formula from Hayes and Bennett [21] for comparing proportions of clustered data. When gravid females were provided with identical treatments in both chambers, 24 replicates resulted in a similar proportion in each chamber (p1 = 0.5). The variability of the nightly catches was used to calculate the coefficient of variation (ratio of standard deviation/mean), which was high at 0.33. Assuming that out of 100 mosquitoes released, 80 respond by entering one or the other collection chamber, 24 replicates in each arm (p1 and p2) can detect an increase or decrease in the catch rate of 20% (p2 = 0.7) with 90% power at a 5% significance level. Data loggers (Tinytag, TV4500) were placed in the two collection chambers and the release compartment for three nights in each of the three treatments to measure relative humidity.

### Statistical analysis

Data were analysed using generalized linear models comparing the mean proportion of female mosquitoes responding to the test cage or the test compartment. Responses of non-fed and gravid females towards the humid cage were compared in WHO-tube bioassays. Odds ratios were calculated in reference to the response of non-gravid females. In the airflow olfactometer bioassays responses of gravid females towards the three different experimental treatments (dry-dry, water-water, dry-water) were compared. Odds ratios were calculated in reference to the wet-wet comparison (equal treatments). The experimental treatments, the olfactometer (A, B, C) and the collection chamber (left, right) were entered as fixed factors to estimate their impact on the outcome. Since the data were highly over-dispersed, quasibinominal distributions were used. Mean proportions per treatment and their 95% CIs were calculated using the parameter estimates of the models by removing the intercept from the models. All analyses were done with R statistical software version 2.14.2 [22].

## Results

### WHO-tube bioassays

**Figure 3:**
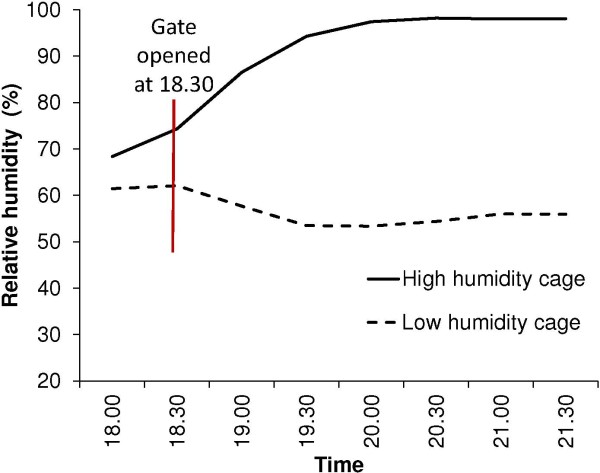
Average humidity in high and low humidity cages in tube bioassays.

At the time when the gates of the WHO tubes were completely opened mean relative humidity differed by around 12% between high and low humidity cages. Humidity slowly decreased in the low cage and increased in the high humidity cage over the next two hours and the difference reached a maximum of approximately 44% at 20.00, with a mean relative humidity of 54% (95% CI 53-56%) in low and 97% (95% CI 95-99%) in high humidity cages (Figure 3). Average temperatures during the experiments ranged between 27 and 28°C. Conditions were similar in both experiments with unfed and gravid females.

**Figure 4:**
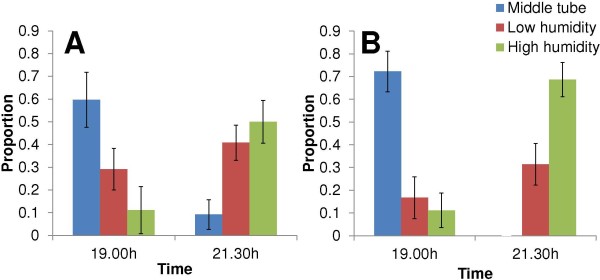
**Mean proportion of females (error bars = 95% confidence intervals) resting in release (middle tube), low or high humidity cage at start and end of experiment with *Anopheles gambiae s.s. *in tube bioassays.** Non-fed females **(A)** and gravid females **(B)**.

At 19.00, half an hour after the gates were opened, 60% of the non-fed mosquitoes and 72% of the gravid mosquitoes remained in the middle tube; 29% of the unfed mosquitoes moved to the low and 11% to the high humidity cages. Gravid females had moved only in small and similar proportions to the low and high humidity cages (Figure 4).

Unfed females showed no preference for any of the two conditions provided (Table 1, Figure 4). When gates were closed at 21.30 half of the unfed females had moved in the high humidity cage and the other half either remained in the middle tube (9%) or moved into the low humidity cage (41%). In contrast, gravid females were 2.4 times more likely to move to the high humidity cage than unfed females (Table 1). All gravid females had moved out of the middle tube at 21.30 and on average 71% of them had moved into the high humidity cage.

**Table 1:** **The mean percentage of gravid *Anopheles gambiae s.s. *** **attracted to the test cage in the WHO-tube bioassays and to the test compartment in the airflow olfactometer bioassays**

**Experimental treatment**	Mean percentage (%) in test (95% CI)*	**Odds ratio (95% CI)**	**p**
**Response towards high humidity cage (test) in WHO-tube bioassays at 21.30**			
Non-fed females	50 (38-62)	1	
Gravid females	71 (59-81)	2.4 (1.3-4.7)	0.018
**Airflow olfactometer bioassays with gravid *An. gambiae s.s. *** **in three experimental treatments**			
Wet (control) vs. wet (test)	56 (48-64)	1	
Dry (control) vs. dry (test)	50 (29-71)	0.8 (0.3-1.9)	0.598
Dry (control) vs. wet (test)	93 (88-96)	10.6 (5.4-20.8)	< 0.001

CI = confidence interval.

*based on model parameter estimates.

### Airflow olfactometer bioassays

Differences in relative humidity between areas with and without water were lower in the airflow olfactometer experiments than in the cage experiments. Relative humidity was on average 20% higher in chambers that contained water than in areas that did not (collection chamber and/or release compartment). Nightly relative humidity in collection chambers containing water was 91% (95% CI 90-92%), the average relative humidity in dry release compartments or dry chambers was 71% (95% CI 69-72%). The temperature did not differ between collection chambers and release compartments irrespective of the treatments and was on average 27.7°C (95% CI 27.2-27.9°C) during the 24 nights of experiments.

**Figure 5:**
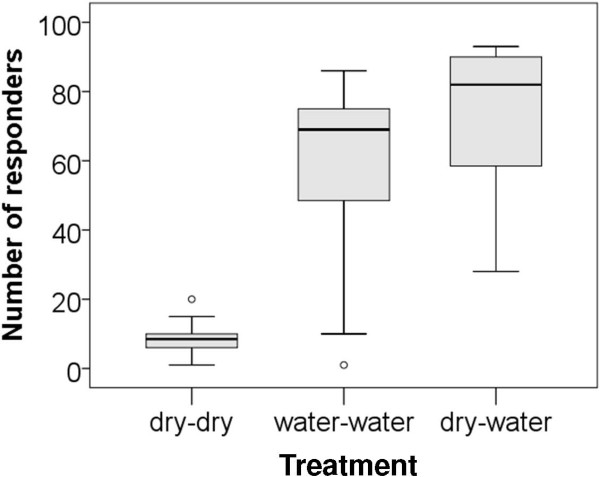
**Comparison of response rates of gravid *Anopheles gambiae s.s. *** **to the three experimental treatments tested in airflow olfactometers.**

High responses of gravid females were recorded in the experimental treatments that presented water in either one or both collection chambers of the olfactometer (median of 69-83%, n = 100 per olfactometer/experimental unit). In contrast, when no stimulus was provided only a median of 9% of the mosquitoes responded by flying upwind in any of the two chambers whilst the rest remained in the release compartment (Figure 5).

When presented with an identical treatment the gravid females approached both collection chambers in equal proportion (estimated ratio 1:1) whilst on average 93% of the gravid females chose the chamber with water (estimated ratio 1:11), when the other was dry (Table 1) irrespective of whether the test cup was presented in the left or right collection chamber and irrespective of which of the three olfactometers was used for the test (both factors were not significantly related to the outcome).

## Discussion

Here evidence is presented that gravid *An. gambiae s.s.* move from lower humidity towards higher humidity. This has been shown at short distances of 15-20 cm in still air and along an air stream of moving water vapour towards an area of higher humidity at longer distances of about 60 cm. Whilst one cannot be certain that gravid females are attracted to water vapour, since they could be repelled from drier areas, it is more likely that attractiveness of water vapour was responsible for the strong results observed since the relative humidity in the low humidity test areas was close to 60% and above, which is similar to the relative humidity of their resting places [23,24]. This is supported by the results with unfed females, which did not show any preference for moving into the higher humidity cage compared to the lower humidity cage. Nevertheless, it has been shown with all physiological stages that individuals can orientate to water vapour plumes or humidity differences much in the same way that a mosquito locates a host [25]. Early studies indicated that in *Aedes aegypti* humidity receptors were present on the antennae of females [26]. In *Anopheles atroparvus* the hygroreceptors were located on the distal segments of the antennae bearing most of the grooved pegs [27]. Recent studies with *An. gambiae s.s.* have confirmed that more than half the grooved pegs on the antennae increase their firing rate in the presence of water vapour and that some respond to low humidity, suggesting that these receptors play a role in humidity perception [28]. Whilst it has been shown that humidity is important for the survival of mosquitoes [29], a clear difference in the behaviour of unfed and gravid females was demonstrated in the presented WHO tube experiments. The strong responses observed in gravid mosquitoes towards moving to areas of very high humidity is likely to increase the reproductive success of females, since they are more likely to find an aquatic habitat that might serve as a potential oviposition site, and would therefore be an adaptive trait selected for in nature.

In the tube bioassay, only a small number of gravid mosquitoes left the central holding tube immediately after the gates were opened. This might indicate that mosquitoes remained static long enough for detecting the humidity differences and direction before moving, especially since the difference in humidity was only around 12% at the time when the gates were opened and no airflow was created. However, at the end of the peak oviposition period 2.4 times more mosquitoes had moved into the humid cage than the drier one whilst the response of unfed females was similar towards the two treatments.

The attraction of water vapour is demonstrated clearly with free-flying mosquitoes in airflow olfactometers. Here seven to eight times more gravid mosquitoes were found in the collection chambers when one or both chambers contain water than when both were dry. Furthermore, when given a choice between one chamber containing water and one that is dry, 11 times more gravid females were collected in the chamber with water. The upwind flight was probably stimulated by moist air. It is most likely that the greater attractiveness of water vapour in a wind tunnel than in the tubes was a result of moving moist air in the tunnel compared with the relatively still air in the tubes. Whilst the evidence presented here shows the attraction of water vapour over relatively short distances, previously published work provides support that water vapour might attract females over several metres. Dugassa *et al.* demonstrated that when gravid *An. gambiae s.s.* females were released into a large screened semi-field system the attractiveness of a reflecting surface was increased by 60% when presented close to water compared with when it was presented without water [30]. In this case females travelled at least 5 m from the release point to the site where they were collected. *Anopheles gambiae* is highly sensitive to subtle changes in moisture as seen when selecting moist sites for ovipositing [31].

It cannot be totally excluded that chemicals other than water were released from the tap water in the experiments described in this paper, since water purification with charcoal-sand filters does not completely sterilize the water or remove all chemicals. Nevertheless, the observed attraction was very strong, especially in the airflow olfactometers. If this was based on semiochemicals released from the tap water, an effect should have been observed to larger degree in the preliminary experiments comparing tap water with double-distilled water. However, in these experiments only a very slight and insignificant preference for the tap water was recorded (Additional file 1).

The present work supports the conclusion made by Kennedy that ‘water vapour emanating from a surface plays an important part in evoking pre-ovipository responses in mosquitoes (*An. atroparvus*, *Ae. aegypti* and *Culex molestus*)’ [32]. He also recognized the importance of moist air currents to activate movement and help with orientation which ‘very probably play an important part in water-finding in the field’. Such conditions existed in the olfactometer experiments. The question arises if and how gravid mosquitoes might use water vapour to navigate through the landscape. The pattern of water vapour across the savanna can be highly heterogeneous, shaped by the local climate, topography, vegetation, soil characteristics and presence and extent of water bodies [33]. The authors are not aware of research that has been conducted that describes the distribution, movement and concentration of water vapour at dusk in the savanna regions of tropical Africa at less than one metre above the ground; the environment encountered by gravid *An. gambiae* searching for a water body in which to lay their eggs. Such research is likely to provide further insights into the pre-oviposition behaviour of this important vector.

Water vapour is likely to be a general attractant for all mosquito species whatever their physiological status and it should not be considered the only attractive compound guiding gravid *An. gambiae s.s.* to an oviposition site. Water vapour has been shown to attract host-seeking mosquitoes [13] and indoor-resting mosquitoes [34]. For host-seeking mosquitoes water vapour can indicate a human host, and for resting mosquitoes it provides an environment where the insect is less likely to dehydrate and die, so increasing its chances of survival. Nevertheless, the results presented here clearly show a difference between the responses of unfed and gravid females towards water vapour suggesting that it is an important cue for a gravid mosquito locating a potential water body, though it clearly cannot be the only one. If it was the only cue mosquitoes would accumulate in large bodies of water like lakes, rivers and seas, habitats inimical to their survival. Water vapour is likely to work in a synergistic manner with visual cues possibly over a longer range [35] and with semiochemicals attracting and repelling gravid *An. gambiae* mosquitoes over short distances [14,15,36,37].

## Conclusion

Gravid malaria vectors need to find suitable water bodies for their aquatic life stages to develop. Water consistently evaporates from aquatic habitats making water vapour probably the major chemical signal emanating from a potential larval habitat. This study demonstrates that gravid *An. gambiae s.s.* move into areas of high humidity or along airstreams of water vapour at the time of night they are actively seeking a site to lay their eggs, implicating water vapour as an important pre-oviposition attractant. More research is needed to address: (1) how water vapour is distributed over the landscape, (2) whether it assists gravid females in locating potential aquatic habitats over longer distances, and, (3) how it interacts with other pre-oviposition cues, either visual or chemical.

## Competing interests

The authors have declared that they have no competing interests.

## Authors’ contributions

SWL, UF, MO, MH, conceived the idea for this research. SWL, BF and MH developed the experimental design for the tube bioassays and implemented them with gravid females; MO implemented them with non-fed females. MO designed and built the airflow olfactometers. MO and UF developed the experimental design and implemented the olfactometer bioassays. MH contributed the additional data comparing distilled water with tap water. MO and UF analysed the data and BF, SWL and UF collated the first manuscript draft. All authors contributed to the final draft, read and approved the manuscript.

## Supplementary Material

Additional file 1 **Cage bioassays comparing the oviposition response of *Anopheles gambiae s.s. *to filtered tap water and distilled water in two choice experiments.** The document presents the background, methods and results of the experiment. Click here for file
