# Supplementary material for: Water vapour is a pre-oviposition attractant for the malaria vector Anopheles gambiae sensu stricto
Source: Malar J. 2013 Oct 11;12:365. doi: 10.1186/1475-2875-12-365 (PMC3907035; doi:10.1186/1475-2875-12-365)
Supplement: Additional file 1 — Cage bioassays comparing the oviposition response of Anopheles gambiae s.s. to filtered tap water and distilled water in two choice experiments. The document presents the background, methods and results of the experiment. [file 1475-2875-12-365-S1.pdf]

Additional file 1:

Cage bioassays comparing the oviposition response of *Anopheles gambiae* s.s. to filtered tap water and distilled water in two choice experiments

Oviposition bioassays need to be replicated in large numbers to account for the variability in responses of gravid females from different egg batches and under different climate conditions. Consequently, a large amount of water is needed as oviposition substrates. Distilled water is frequently used in oviposition bioassays but can be a limiting factor when working at remote field sites. The authors therefore aimed to evaluate whether purified lake water can be used as alternative to distilled water in oviposition bioassays.

Methods:

Piped non-chlorinated water pumped from Lake Victoria was passed slowly through a sand charcoal gravel filter for purification (referred to as filtered tap water). The aim was to remove large and small particles from the water including the majority of algae and bacteria. Two choice cage bioassays were carried out comparing the oviposition response of 300 individual gravid *Anopheles gambiae s.s.* females to filtered tap water versus double-distilled water. Bioassays were done in 30×30×30 cm cages. The cages had a steel framework founded on a galvanized metallic base and covered with fine mosquito netting. The cage-net also had an insert sleeve for introducing and retrieving oviposition substrates and gravid mosquitoes. Oviposition substrates were offered in 70 mm diameter glass cups (Pyrex®) that were autoclaved and afterwards kept in an oven at 200°C for at least 2 hours before experiments. In each cage two cups were provided in opposite corners one filled with 100 ml filtered tap water (test) and the other cup filled with 100 ml double-distilled water (control). The arrangement of oviposition cups was systematically altered between adjacent cages to

adjust for position effect. The test cup was randomly placed in one corner of the first cage and test cups in subsequent cages were moved one corner step in a clockwise direction relative to that of the preceding cup. Corresponding control cups were added in each cage diagonal to the test cup to complete a two choice set up. Adjacent cages were placed on a table a minimum of 30 cm apart. The experiment was carried out under ambient light and temperature conditions in makeshift huts. Two huts were used containing two tables each. Twenty-five cages were placed per table totalling 100 cages per experimental nights. The experiment was replicated for three rounds using different batches of mosquitoes. Individual mosquitoes were placed in the cages at 18:00h and the response (presence of eggs) per treatment and cage recorded at 8:00h in the morning.

Data analysis:

Data was analysed with R statistical software version 2.14.2 using the one sample proportions test with continuity correction. This test investigates whether the response rate of individual gravid females towards the two treatments differs significantly from 0.5 hypothesizing that if the two treatments would be equally suitable for oviposition 50% of the females should have laid in the test and 50% in the control.

Results:

In total 242 out of the 300 females laid eggs (81%). Out of the 242 females 33 (14%) laid in both test and control cups. The remaining 209 females laid either in test or control. Therefore in total 275 responses were recorded. The bioassays were carried out in three rounds. Table S1 shows the results of the proportion tests for the individual rounds and for the pooled data.

Table S1: Response rate of gravid females towards the filtered tap water

	Number of responses (n/N)	Response rate (%) for filtered tap water (95% CI)	p-value*
**Round 1**	59/105	56 (46-66)	0.242
**Round 2**	50/89	56 (45-67)	0.289
**Round 3**	45/81	56 (44-66)	0.341
**TOTAL**	**154/275**	**56 (49-62)**	**0.054**

*null hypothesis: response rate equals 50%

The test cup with the filtered tap water received 56% of all responses (eggs laid) towards the two treatments both in the individual rounds as well as when all data were pooled for analyses. This only slightly increased proportion was neither significantly different from 50% for the individual rounds nor when the data were pooled at the ≤0.05 significance level. Nevertheless, when the data were pooled the difference approached significance.

Conclusion:

Gravid *An. gambiae* s.s. females did not show any strong preference for the filtered tap water over the double-distilled water. The approaching significance level when the data were pooled might indicate a genuine effect likely reflecting a difference in water quality due to the incomplete filtration of algae and bacteria from the water. Nevertheless, the difference between the two water sources was so small that the authors conclude that the filtered tap water does not contain strong oviposition semiochemicals and can be used for studying the oviposition behavior towards chemical and visual cues as a replacement for distilled water.
